# Exploring a Nursing Home-Specific, Interdisciplinary, Function-Focused, Communicative Framework Based on Situation, Background, Assessment, and Recommendation

**DOI:** 10.1097/jnr.0000000000000428

**Published:** 2021-04-09

**Authors:** Min Sun PARK, Su Jung LEE, Young Rim CHOI, Sung Ok CHANG

**Affiliations:** 1 PhD, RN, Assistant Professor, Department of Nursing, Cheongju University, Cheongju, Republic of Korea; 2 PhD, RN, Research Professor, College of Nursing, Korea University, Seoul, Republic of Korea; 3 PhD, RN, Professor, College of Nursing, and BK21 FOUR R&E Center for Learning Health Systems, Korea University, Seoul, Republic of Korea.

**Keywords:** older adults, communication, interdisciplinary studies, nursing homes, SBAR

## Abstract

**Background:**

Improved methods of communication are needed among professionals in related fields to address the increasing complexity of clinical situations and various levels of functioning experienced by older adults who live in nursing homes.

**Purpose:**

The purpose of this study was to explore function-focused clinical communication among nurses and providers based on the Situation, Background, Assessment, Recommendation (SBAR) approach toward interdisciplinary collaboration to maintain function among nursing home residents and to identify the characteristics of SBAR flows in nursing homes.

**Methods:**

Detailed interviews with 28 interdisciplinary professionals working in four nursing homes were conducted. Directed qualitative content analysis was used to identify the internal attributes of SBAR-based communication. Case analysis was conducted to identify SBAR flows.

**Results:**

Four themes emerged as key factors for function-focused interdisciplinary staff communication in nursing homes. Effective nursing care to maintain function among nursing home residents requires accurate awareness of abnormal circumstances. Knowledge of assessment and resident background are needed to address situations requiring intervention and identify the problems underlying a resident's current state. The optimal therapeutic environment is created by sharing roles and tasks among practitioners through referrals.

Twelve generalized situations requiring function-focused communication (i.e., dislocation of body line because of joint contracture, change in walking, difficulty of moving because of pain, difficulty in eating, fever, change in sleep pattern, change in excretion pattern, change in weight, change in condition, change in problematic behavior, decrease in cognitive function, and change in relationships) and the related nurse-centered SBAR pathways were identified.

**Conclusions/Implications for Practice:**

These results represent a first prototype for developing practical communication guidelines for nursing-home-specific function-focused care and provide new insights into the interdisciplinary approach.

## Introduction

As geriatric syndromes have multiple causes and underlying diseases, assessment and intervention require input from multiple experts in related fields (Colón-Emeric et al., 2017). Increased clinical complexity is difficult to manage with the application of only a single domain of expertise and instead requires responses based on interdisciplinary team consensus and cooperation (Colón-Emeric et al., 2017; Renz & Carrington, 2016). Nursing homes are residential long-term care facilities in the community that rely on the constant coordination of multiple disciplines. Residents comprise older individuals who experience dementia and whose function is impaired by two or more complications (Lee et al., 2019; Williams et al., 2018). Previous research on interdisciplinary collaboration in the nursing home setting revealed that providing care to residents who might have experienced fractures requires the synthesis of judgments of interdisciplinary teams based on nurses' medical knowledge of osteoporosis, physical therapists' knowledge of range of motion (ROM), and social workers' knowledge of social isolation (Park, Kim, et al., 2019; Park, Lee, & Chang, 2019). Therefore, developing ways to improve communication among interdisciplinary health professionals is key to improved quality of nursing home care (Alexander, 2015).

Proper communication among related professionals when providing function management has a direct impact on care outcomes for patients with dementia (Shaw et al., 2018; Townsend-Gervis et al., 2014). In addition, effective information sharing within the team avoids duplicate interventions, thereby reducing wasted practitioner time and effort during the care process (Xyrichis et al., 2018). In particular, the quality of connections among practitioners, the degree of information flow, and the various perspectives involved in planning care are the key factors that determine care outcomes (Colón-Emeric et al., 2017; Renz & Carrington, 2016). Thus, observations of subtle functional changes in older adults should be shared within interdisciplinary teams and cooperatively interpreted for the care plan (Colón-Emeric et al., 2017; Park, Lee, & Chang, 2019). However, previous research and nursing home practice have not emphasized care processes based on such cooperative intercommunication.

The Situation, Background, Assessment, Recommendation (SBAR) tool has been recommended by the World Health Organization to promote standardized, structured communication (Institute for Healthcare Improvement, 2019). The SBAR is a handoff tool that organizes information clearly and concisely to facilitate collaborative communication (Panesar et al., 2016). The SBAR is regarded as a clear and safe communication method for problem solving among different disciplines in the medical environment and a simple way of sharing patient information with other medical professionals (Ashcraft & Owen, 2017; Cornell et al., 2013). The SBAR was developed for use in urgent and time-sensitive medical situations (Thomas et al., 2009; Townsend-Gervis et al., 2014). Thus, research to date has focused mainly on nurse–physician and nurse–nurse interactions, with little literature related to prolonged communication across various fields based on interdisciplinary approaches (Griffiths et al., 2014; Renz & Carrington, 2016; Townsend-Gervis et al., 2014). Thus, the SBAR should be extended to the nursing home environment. In contrast to the urgent clinical situations in general hospitals, nursing homes require sustained sharing of clinical information among professionals to provide appropriate care.

In fact, nursing home care differs slightly from physician-centered, general hospital level care, which focuses on comprehensive professional medical services. To maximize and preserve function in older adult residents, nurses should play a key practitioner role (H. J. Kim et al., 2016; S. J. Kim et al., 2014). In this context, beyond the dichotomous communication between nurses and physicians, communication occurs among nurses and practitioners to find the best solutions to address functional problems of residents. Furthermore, to develop an appropriate SBAR, it is essential to understand how interdisciplinary practitioners make decisions, what assessments and interventions they use, and what they typically refer to other disciplines. The purpose of this study was to explore nurses' and related practitioners' communications about the clinical care necessary to maintain function in nursing home residents and to explore the characteristics of SBAR flows in nursing home settings.

## Methods

The first step was a directed qualitative content analysis to extract the internal attributes of the core concept based on the SBAR framework. The second step was an SBAR-based case analysis to further understand these information flows and identify how information is shared within an interdisciplinary nursing home team.

### Data Collection

Data were collected from August to November 2017. Because the practical knowledge and accumulated know-how gained from the actual experience of practitioners may be used to determine the quality of data obtained through the interviews, the researchers used a purposive sampling method to acquire sufficient data until theoretical saturation was reached. Using semistructured questionnaires, each interview was conducted 1–2 times and was audio recorded. Data were collected using one-on-one interviews with 28 staff members working in four nursing homes of different size categories. The interviewees consisted of seven nurses, six social workers, five physical therapists, one occupational therapist, four nutritionists, and five care helpers. Average participant age was 46 years, ranging from 27 to 63 years, and the average length of experience providing care in nursing homes was 6 years.

The in-depth interviews began with general, open-ended questions about the interdisciplinary approach to care that focused on function in nursing home residents (e.g., “Please explain to me the collaborative approach among related occupations in your nursing home when providing care to improve the quality of life of resident by managing their functions.”). After a general question, the researcher asked questions focusing on the four stages of SBAR, a predetermined category, which probed the practitioners' experience and knowledge of background, assessment, and recommendation in situations where function management requires interdisciplinary collaborative approaches. Furthermore, the researchers asked the staff about referring function-related situations to the relevant practitioners to initiate communication.

### Data Analysis

#### Step 1: A directed qualitative content analysis

A directed content analysis approach, a more structured process than conventional content analysis, was undertaken to verify the developed theoretical framework and expand knowledge and understanding of phenomena that have not been actively studied (Hsieh & Shannon, 2005). The researcher became familiar with the interview data by reading the transcript several times. The researcher reviewed the interview data to identify repeated thoughts, ideas, and phrases, and codes were generated to represent these meaningful concepts. Using the existing framework, the researcher began to analyze the key concepts and variables as the initial coding categories. In this study, four categories of SBAR were used as an initial framework to explore the stages of interdisciplinary communication in nursing homes. The researchers repeatedly reviewed and revised the coded data through discussions within the research team, with the results used as the themes and subthemes (Hsieh & Shannon, 2005).

#### Step 2: Case analysis for identifying Situation, Background, Assessment, Recommendation flows in nursing homes

The SBAR-based case analysis was conducted to explore how information is transmitted among interdisciplinary practitioners in nursing homes and to clarify the relationships between the various disciplines. In the interviews, 39 cases of SBAR were collected and analyzed, and each was categorized into the four stages of SBAR. The professional in the referring role, the professional in the receiving role, and the information shared between them were analyzed.

### Data Credibility

Padgett's (1998) strategy was used to assess the reliability and rigor of the data and the processes of the qualitative research. The researchers were experienced in qualitative research on older adults, were familiar with nursing home facilities, and had long-term contacts with practitioners in nursing homes. All research data were collected via audio recordings, and the transcripts were double-checked with other researchers to ensure reliability. The researchers conducted constant communication and verification with their peers to avoid analysis bias and compared the themes and subthemes they had respectively extracted. The analysis procedures and methods for all of the qualitative studies were undertaken under the supervision of another qualitative researcher, and the final results were verified and evaluated by a qualitative expert with extensive research experience.

### Ethical Considerations

The research began after approval by the institutional review board of the affiliated university (1040548-KU-IRB-17-122-A-1). The researchers also received permission from the participating facilities and obtained written informed consent from participants, who were informed of their autonomy in participation and their right to withdraw from the study at any time.

## Results

### Step 1: A Directed Qualitative Content Analysis

The directed qualitative content analysis of the interview revealed four themes related to the nature and content of SBAR regarding the function-focused care approach among interdisciplinary practitioners in nursing homes and suggested practical communicative care strategies based on the interdisciplinary assessments and interventions in actual nursing home circumstances (Table 1).

**Table 1.** Emerged Themes and Subthemes

No.	Theme	Subtheme
Theme 1	Situation: Recognizing abnormal resident situations/circumstances	1. Gradual deterioration in function
		2. Subtle changes in function
Theme 2	Background: Tracking information related to situations requiring intervention	1. Usual functional status and life patterns 2. Related past medical history
Theme 3	Assessment: Identifying practical issues that practitioners should check in relation to the current situation	1. Functional degradation compared with the past 2. Newly detected unusual symptoms or abnormal behaviors 3. Changed input or intervention 4. Recent unusual events
Theme 4	Recommendation: Sharing the roles and tasks of each practitioner	1. Requesting other occupations for assessments and interventions based on their distinct knowledge 2. Suggesting a better way to improve the quality of life of residents to other practitioners

#### Theme 1. Situation: Recognizing abnormal resident situations/circumstances

Recurring situations relevant to SBAR were extracted from the interviews in the form of situational phenomena requiring interdisciplinary intervention in the practice dimension. In these situations, unusual interventions by interdisciplinary staff who provide direct care to residents were inevitable, and the participants mentioned that the ability of the practitioner to recognize the associated problematic situations was an important factor. The complex subthemes for which functional intervention is recommended included situations of both gradual deterioration and subtle changes in functioning.

1. **Gradual deterioration in function.** In particular, the gradual progression of joint contracture could cause problems for both patients and providers: “*After entering nursing homes, there was an elderly resident whose legs became progressively more rigid. Now it is difficult for them to bend or move joints by loosening legs bent in an X-shape. The nurse recognized that there was a possibility of a fracture occurring when the resident’s joints had to be moved, such as when providing positional change or diaper change*” (a nurse with 11 years of experience).
2. **Subtle changes in function.** In particular, a care helper who provided 24-hour close assistance to the resident would often perceive subtle functional changes when the resident was walking or eating: “*There was a resident who liked walking as an exercise. When I observed him from behind a few days ago, his walking posture seemed a little different from normal and I felt something strange. He limped and did not want to walk. Also, he seemed to walk with his hips pulled back*” (a care helper with 10 years of experience).

#### Theme 2. Background: Tracking information related to situations requiring intervention

Based on SBAR, the participants reported that the background for interventions was compiled from past and present information about residents; this information helped practitioners identify problems requiring functional intervention. In other words, the background corresponded to the knowledge dimension. In addition, practitioners commonly stated that there might be differences in the degree to which appropriate information could be collected, depending on the level of awareness of the problem. Subthemes derived from actual experience in nursing homes consisted of the usual functional status and life patterns of residents related to their past medical history.

1. **Usual functional status and life patterns.** Practitioners stated that it was necessary to focus on symptoms of aging including functional discomfort, which helped them better understand residents' normal functional conditions and lifestyle patterns. Factors to be specifically identified included physical and cognitive functional status, habits (sleep, excretion, and dietary patterns), preferences, responses to care (the degree of cooperation with practitioners), and recent condition: “*There was a resident who enjoyed walking around the unit three times every morning. I used to check his physical function carefully if he tried to rest instead of undertaking his normal walking exercise*” (a care helper with 10 years of experience).
2. **Related past medical history.** Most practitioners noted that underlying diseases (dementia, Parkinson's disease, osteoporosis) and past surgical history (hip replacement) could be key background information, as these may have a continuing impact on cognitive and physical function: “*Operations such as hip arthroplasty can damage the overall functions of the resident. Rehabilitation such as walking exercise to recover physical functions is necessary for them, and changes in cognitive functions should also be checked*” (a nurse with 10 years of experience).

#### Theme 3. Assessment: Identifying practical issues that practitioners should check in relation to the current situation

In the interviews, it was found that, based on SBAR, assessments are evaluations of elements related to problematic situations that require verification by practitioners. In nursing-home-based function-focused care, the assessed elements represented the extent to which changes in life patterns or symptoms occurred in activities of daily living. The practitioners reported that level of assessment may change according to information gathered about a problematic situation. Thus, it could be said that, in the SBAR framework, the theme of assessment also belongs in the knowledge dimension. The subthemes that emerged relevant to identifying functional changes among residents at the assessment stage were as follows:

1. **Functional degradation compared with the past.** Practitioners who observe the daily life of residents are able to evaluate the degree of change in ROM, walking, and the amount of food eaten, although they may have difficulty applying accurate numerical indicators to these variables: “*When I evaluated the range of motion (ROM) of both knees, the elderly resident shouted that he was sick and that his bent knee joints could not be moved at all*” (a nurse with 11 years of experience) and “*During the assisted meal, when the resident ate food, much saliva flowed out of his mouth, and more than half of his food came out of his mouth*” (a social worker with 11 years of experience).
2. **Newly detected unusual symptoms or abnormal behaviors.** Participants reported the necessity of observation not only of physical function, such as patterns of excretion and sleep, but also of cognitive function, such as changes in memory and problematic behavior: “*The resident had walked out of the bed about 3–4 times in the last 3 days, wanting to go to the restroom all night*” (a care helper with 10 years of experience) and “*The resident was angry and showed problematic behavior such as violent language and threatening behavior in the ward*” (a social worker with 4 years of experience).
3. **Changed input or intervention**. Practitioners must assess changes in medication type and dosage, diet and snacks, and the types and timing of provided programs: “*Recently, the resident has not eaten much more than half of the provided meals even though I changed his diet from rice to porridge*” (a nutritionist with 2 years of experience).
4. **Recent unusual events.** Practitioners noted that changes in physical and cognitive function as well as changes in social functions such as relationships with family and practitioners or worrying events should not be overlooked: “*A few days ago, there was a conflict between two residents because of where they were seated in the program. They sat next to each other and continued their fighting. The social worker separated them to stop their fight*” (a social worker with 3 years of experience).

#### Theme 4. Recommendation: Sharing the roles and tasks of each practitioner

The recommendation based on the SBAR was to divide tasks among the related professions and share appropriate care when referring to other practitioners in the interdisciplinary team to develop the most appropriate care plan. The ultimate goal of practitioners' interactions was the best care outcomes and management of function for nursing home residents; this was achieved by synthesizing the professional interventions of each discipline through the exchange of requests and suggestions (Table 2). These elements, which belonged to the share and distribution dimension of interdisciplinary communication, included requesting other occupations for assessments and interventions based on their distinct knowledge and suggesting to other practitioners better ways to improve the quality of life of residents.

**Table 2.** Extracted Practice Strategies Expected in Function-Focused Referral Situations in SBAR

Function-Focused Situation	Sending Position	Accepting Position	Practice Strategies Expected When Referring to Another Discipline
Dislocation of body line because of joint contracture	NS	PT	Checking safe range of motion
Change in walking	NS	PT	Checking safe range of walking exercises
	CH	NS	Assessing physical functions based on medical knowledge (fracture, swelling, etc.)
	PT	NS	Assessing pain and requesting hospital care
	PT	CH	Educating residents on how to walk safely in daily activities
Difficulty of moving because of pain	NS	PT	Relieving pain
Difficulty of eating	NS	NT	Providing an alternative or preferred diet
	CH	NS	Checking changes in the mouth Assessing swallowing and chewing problems
	CH	SW	Asking the family to make the resident's favorite food
	CH	NT	Providing proper snacks and implementing a proper diet
	OT	NS	Checking current medications
	OT	NT	Providing an alternative diet
	OT	CH	Providing proper assistance during meals
	OT	SW	Considering the resident's preferred colors when providing programs
	SW	NS	Assessing oral wounds as well as teeth and chewing problems
	SW	OT	Assessing oral wounds as well as teeth and chewing problems
	SW	NT	Requesting a proper diet
	CH	NS	Assessing chewing problems
	CH	NS	Assessing digestive problems
	NT	NS	Assessing chewing problems
	NT	CH	Continuous observing and reporting during meal assistance
Fever	CH	NS	Assessing physical functions based on medical knowledge
Change in sleep pattern	CH	NS	Intervening with medication and requesting hospital care
Change in excretion pattern	NS	CH	Observing and reporting urinary symptoms and urine patterns
	CH	NS	Intervening with medication
	NS	NT	Changing diet
Change in weight	NT	NS	Identifying increases and decreases in body weight
	NT	SW	Managing nutritional status and requesting nutritional support
Change in condition	PT	NS	Checking vital signs at the time of problem
	SW	NS	Checking vital signs at the time of problem
	SW	CH	Continuous observing and reporting of the resident's condition
Change in problematic behavior	SW	NS	Checking changes in behavior patterns Encouraging the resident to go the hospital and take medication
	CH	NS	Intervening with medication
	SW	NS	Considering hospitalization Writing up behavioral patterns
Decrease in cognitive function	NS	PT	Assessing fall risk Providing physical therapy in a place suitable for physical function
	SW	CH	Continuous observing and reporting on changes in activities of daily living and changes in cognitive functions
	SW	NS	Checking changes in cognitive functions Requesting a focus case conference
Change in relationships	SW	CH	Observing and reporting relationships with the surrounding older adults
	SW	NS	Requesting coordination so that problems do not occur

***Note.*** SBAR = Situation, Background, Assessment, Recommendation; NS = nurse; PT = physical therapist; OT = occupational therapist; SW = social worker; NT = nutritionist; CH = care helper.

1. **Requesting other occupations for assessments and interventions based on their distinct knowledge.** Practitioners generally requested more in-depth assessments and interventions based on the expertise of other disciplines or requested constant monitoring and reporting of a problematic situation: “*The occupational therapist requested the nurse to check for any recently changed medications or doses*” (an occupational therapist with 2 years of experience) and “*In the program, I noticed that the condition of the resident had deteriorated. Thus, I requested the care helper to continuously observe the resident in the ward and to promptly report any problems to the nurse*” (a social worker with 1 year of experience).
2. **Suggesting a better way to improve the quality of life of residents to other practitioners.** Practitioners suggested interventions to improve the quality of daily life for nursing home residents that did not occur to other occupational groups: “*If the resident has a large number of medications to take, the occupational therapist will suggest the nurse to divide the appropriate number of medications by type of drug before and after meals*” (an occupational therapist with 2 years of experience) and “*The nurse suggested that the physical therapist identify ways of minimizing damage to the joints and the extent of the safe range of motion, even when residents cannot move their joints within the normal range*” (a nurse with 11 years of experience).

### Step 2: Case Analysis for Identifying Situation, Background, Assessment, Recommendation Flows in Nursing Homes

From the case analysis for identifying SBAR flows in nursing homes, the researchers extracted 39 SBAR cases related to interdisciplinary function-focused communications from the 28 practitioner interviews. In addition, the researchers classified the general function-focused communications of the 39 SBAR cases into 12 categories. The left vertical row in Table 2 presents 12 general categories of function-focused situation requiring interdisciplinary communication. In SBAR, the communication situation addressing “difficulty of eating” was found to be the most common, corresponding to 15 of the 39 SBAR cases. The next most common general function-focused communicative situation, “change in walking,” corresponded to four of the 39 SBAR cases (Table 2).

The results of the case analysis of the positions of senders and acceptors based on SBAR are shown in Table 2. Senders were generally practitioners who determined that situations required functional intervention and requested input from relevant occupations. Of the 39 SBAR cases, the sender was a social worker in 11 cases, a care helper in 10 cases, and a nurse in seven cases. Social workers and care helpers asked nurses for assessments based on professional medical knowledge. Nurses asked physical therapists mainly about walking, ROM, and pain. Acceptors were generally practitioners who were able to advise with expertise beyond the sender's level of care, provide more in-depth assessments and interventions, and observe subtle changes to solve the problem. Of the 39 SBAR cases, the accepting practitioner was a nurse in 17 cases and a care helper in seven cases.

The results of the SBAR case analysis of the interdisciplinary practice strategies expected on referral to other disciplines are shown in Table 2. Nurses were expected to evaluate functional status primarily based on medical knowledge. Example observations that reflected SBAR communication included weight gain and loss, wounds in the mouth, fractures, swelling, pain, digestion, excretion, medications, and hospital care. Care helpers were expected to observe and report on residents' actual condition and activities of daily living. The physical therapist was expected to employ knowledge about physical movement such as ROM, strength, pain, and fall risk. The occupational therapist was expected to employ practical strategies that made the daily activities of residents more comfortable through stimulation of physical and cognitive sensations. Social workers were expected to employ knowledge about a resident's internal and external resources such as personality and preference, key related people, family relationships, and economic support. Nutritionists were expected to employ their knowledge of eating such as appropriate and preferred diets and eating problems. Representative examples of nursing-home-specific interdisciplinary function-focused communications based on SBAR are presented in Table 3.

**Table 3.** Representative Examples of Nursing-Home-Specific Interdisciplinary Function-Focused Communications Based on SBAR

Function-Focused Situation/SBAR	Sample
Changes in walking	
Situation	*When I (physical therapist) looked at the back of the resident walking, one foot was everted. Compared to usual, today she seemed to walk uncomfortably and bent over her waist a lot.*
Background	*Cognitively, the elderly had enough cognitive ability to communicate and was good at expressing pain. Physically, the resident walked independently using a walker. She had the habit of sitting down sufficiently hard in a wheelchair making a noise.*
Assessment	*I stopped her walking to check if she was able to stand, and she could stand alone for five minutes. I took the resident to her bed and asked where the pain was, and she complained of back pain.*
Recommendation	1. The physical therapist informs the nurse that the resident's physical condition is worse than before and requests hospital treatment if the resident continues to experience back pain. 2. The physical therapist also requests the care helper to assist the resident to sit down slowly when sitting in a wheelchair.
Difficulty of eating	
Situation	*When I (care helper) recently helped feeding the elderly person, I found that he was worse than usual and left much more food than before. I was worried that nutritional problems would arise if he was continuously unable to eat.*
Background	*Since, usually the elderly had a big appetite and could eat a general diet, the elderly had been judged as having no particular problems with their digestive function.*
Assessment	*The weight of the elderly decreased by 3 kg compared to the previous month. When I looked inside his mouth, I saw a scar, which seemed to be a little torn and tingling.*
Recommendation	1. The care helper asked the nurse to provide an appropriate ointment that can be applied in the mouth. 2. The care helper asked the social worker to ask the family member to bring food that the resident likes. 3. The care helper asked the nutritionist to provide the resident with adequate snacks to increase their weight. If necessary, the care helper will request the nurse and nutritionist for a dietary change from general meals to a liquid diet.

***Note.*** SBAR = Situation, Background, Assessment, Recommendation.

**Figure 1.**
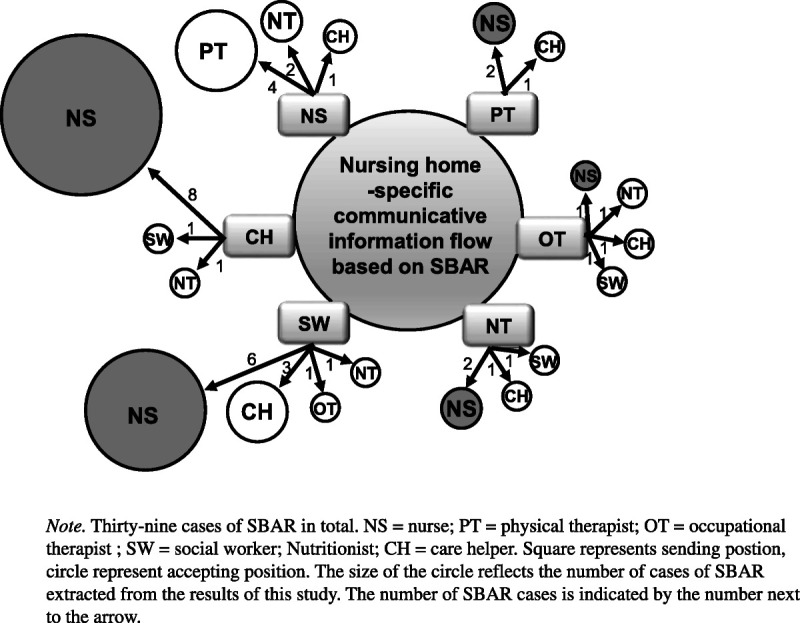
Situation, Background, Assessment, Recommendation (SBAR) flows of nursing-home-specific interdisciplinary function-focused communication

Flows of nursing-home-specific interdisciplinary function-focused communication in terms of the number of SBAR cases based on Table 2 are presented in Figure 1. Of the 39 SBAR cases, the highest number (eight) involved care helpers asking the nurses to communicate. The next highest number of cases were social workers asking nurses (six), followed by nurses asking physical therapists (four).

## Discussion

To the best of the authors' knowledge, this study is the first attempt to explore interactive communication in the context of interdisciplinary function-focused approaches in nursing homes conducted by identifying the nurse and related practitioners' clinical communications and interpreting the characteristics of the SBAR flows. Thus, this study provides new perspectives on nursing home interdisciplinary communications in areas including exchanges of specialized and common knowledge and the transfer of information on welfare in daily life, proper diet, and rehabilitation, which spans different yet related fields, as well as perspectives on nursing in nursing home settings, as opposed to hospital settings, which focus more on disease treatment.

Overall, the four themes developed here proposed communicative strategies that are practical and available in current clinical practice and based on the characteristics of the interdisciplinary approach specific to the nursing home context, which differs from that of general hospitals. Moreover, the results highlight the necessity of continuous connectivity among the practice, knowledge, and share and distribution dimensions based on continuous relationships among interdisciplinary practitioners. In particular, 12 general situations requiring function-focused intervention were extracted, with the recommendation that practitioners should be able to communicate with each other interactively if such problems develop (Resnick et al., 2013). From this perspective, there was a similar discussion in the previous study on the importance of practitioners' abundant real-world experience in the “practice domain” for the development of effective practical care strategies (H. S. Kim, 2010; Park et al., 2018).

In addition, the results of the analysis of the SBAR flows covered all SBAR cases extracted from the interviews, enabling the clarification of entire work roles of related practitioners at a glance, and provided practice strategies for coordinating residents' care plans at each step with other disciplines (Park, Kim, et al., 2019). As shown in Table 2, the number of nurses (19) in the accepting position was the highest by far, followed by care helpers (7), nutritionists (5), physical therapists (4), and social workers (3). On the other hand, the sending position featured similar numbers of social workers (11), care helpers (10), and nurses (7). This finding implies that interdisciplinary practitioners in nursing homes may determine and prioritize problems and set care plans using assessments based on professional nurse-centered medical knowledge. In particular, as the target residents of nursing homes are those older than 80 years old with various symptoms associated with multicomplex underlying diseases, including impaired physical and cognitive function, obtaining the baseline assessment of nurses is indispensable (Asadi et al., 2019; Park, Kim, et al., 2019; Park, Lee, & Chang, 2019; Shin, 2019). As shown in Figure 1, a pathway of communication involving the nurse may be derived when interdisciplinary practitioners communicate in nursing home settings. This result is consistent with the role of nurses as key practitioners and core information providers in nursing homes, as supported in previous studies (Park, Kim, et al., 2019; Park, Lee, & Chang, 2019; Park et al., 2018).

As nursing homes are complex adaptive systems in which interdisciplinary practitioners maintain continuous two-way communications, research on related intercommunication activities has been difficult until recently (Anderson et al., 2003; Griffiths et al., 2014). For this reason, there has been a need to explore the information exchange and information transfers that occur among a variety of related interdisciplinary areas beyond those between nurses and physicians or between nurses and the nursing team. Thus, the new framework of communication derived in this study suggested a prototype platform for the development of standardized SBAR in community nursing home settings by identifying effective communicative examples and patterns among interdisciplinary practitioners, including professional care, rehabilitation, and nursing.

This study was affected by several limitations. First, although the numbers of facilities (4) and interdisciplinary practitioners (28) participating in this study were relatively small, this study, which targeted practitioners providing care to approximately 500 older adult residents, is significant considering its wide-ranging diversity in terms of the sizes and characteristics of the facilities included. In addition, researchers tried to generalize the final 39 cases by eliminating redundant function-focused communicative cases derived from more than 30 in-depth interviews with interdisciplinary practitioners. Finally, the researchers suggest that further research into interdisciplinary practical communicative knowledge may be refined by studying more facilities, particularly in areas with differing cultural backgrounds.

### Conclusions

The results of this research support the idea that, in nursing home practice, the quality of interactive communications between interdisciplinary professionals is based on an external communicative framework and internal information flows. In addition, the extracted SBAR framework and pathways of information flow may be applied when teaching communication methods to nurses and related practitioners in long-term care facilities to improve the quality of interdisciplinary collaborative care focused on managing function among residents. This method of communication regarding residents who complain of multiple discomforts in nursing homes should avoid dichotomies of communication between different occupations; transform them to provide optimal interventions, especially for top-priority problems; and be based on collaborative consensus among all related practitioners. The original framework of nursing-home-specific interdisciplinary communication developed here may be used as a guide containing representative communicative examples for new practitioners in various fields, including beginner nurses, and may serve as a first prototype for developing interdisciplinary communicative guidelines focused on the various needs and discomforts of older adults living in nursing homes.
